# A neutrally stable shell in a Stokes flow: a rotational Taylor's sheet

**DOI:** 10.1098/rspa.2019.0178

**Published:** 2019-07-31

**Authors:** G. orsi, A. De Simone, C. Maurini, S. Vidoli

**Affiliations:** 1 SISSA-International School for Advanced Studies, Via Bonomea 265, 34136 Trieste, Italy; 2 The BioRobotics Institute, Scuola Superiore Sant'Anna, 56127 Pisa, Italy; 3 CNRS, Institut Jean Le Rond d'Alembert, Sorbonne Université, UMR 7190, 75005, Paris, France; 4 Dip. Ingegneria Strutturale e Geotecnica, Sapienza Università di Roma, via Eudossiana 18, 00184 Rome, Italy

**Keywords:** viscous flows, micromotility, morphing shells

## Abstract

In a seminal paper published in 1951, Taylor studied the interactions between a viscous fluid and an immersed flat sheet which is subjected to a travelling wave of transversal displacement. The net reaction of the fluid over the sheet turned out to be a force in the direction of the wave phase-speed. This effect is a key mechanism for the swimming of micro-organisms in viscous fluids. Here, we study the interaction between a viscous fluid and a special class of nonlinear morphing shells. We consider pre-stressed shells showing a one-dimensional set of neutrally stable equilibria with almost cylindrical configurations. Their shape can be effectively controlled through embedded active materials, generating a large-amplitude shape-wave associated with precession of the axis of maximal curvature. We show that this shape-wave constitutes the rotational analogue of a Taylor's sheet, where the translational swimming velocity is replaced by an angular velocity. Despite the net force acting on the shell vanishes, the resultant torque does not. A similar mechanism can be used to manoeuver in viscous fluids.

## 1. Introduction

The problem of locomotion at low Reynolds numbers has been initiated by the groundbreaking paper of Taylor [1], which has had an enormous impact and continue to motivate a substantial amount of research (e.g. [2] and references cited therein, and the more than 1000 references that have cited this review paper since its publication 10 years ago).

The model proposed by Taylor is one of the simplest (and yet enlightening) examples of swimming through low Reynolds number flows that can be treated analytically. In [1], he considered the self-propulsion mechanism of a two-dimensional sheet, immersed in a viscous fluid, on which waves of transversal displacement propagate. Assuming these waves have small amplitude, a perturbative expansion of the boundary conditions can be used to compute the swimming speed of the oscillating sheet. In more detail, if an unbounded fluid is considered, and the undeformed sheet coincides with the plane *y* = 0, the travelling wave propagating in the *x*-direction will cause a vertical displacement

1.1 $$y_{0}=b\sin(k(x-vt)),$$

where *b* is the amplitude, *k* the wavenumber and *v* is the wave phase speed. In a reference frame moving with the sheet, the boundary condition on the velocity of the fluid in contact with the sheet [2] will be

1.2 $$u(x,y_{0}(x,t))=-bkv\cos(k(x-vt))e_{y},$$

while it is expected that infinitely far from the sheet a uniform and steady flow will be observed

1.3 $$\lim_{y\to \infty }u(x,y)=-Ue_{x},$$

where *U* is the swimming speed of the sheet. With this choice of frame, the swimming speed appears as an unknown boundary condition for the problem. Given the assumption of small amplitude, Taylor shows that expanding the boundary condition (1.2) in powers of the dimensionless parameter *bk*, and knowing the form of the general solution for the two-dimensional Stokes flow, one can solve the problem approximating *U* with an increasing order of accuracy. The problem, as posed, is therefore closed even if the unknown appears as a boundary condition. Up to second-order terms in *b* *k*, this velocity is

1.4 $$U=-\frac{1}{2}v{\left(bk\right)}^{2}.$$

Taylor's analysis can be used to show that, when deformations are small, travelling waves of bending (respectively, stretching) cause translation in the direction opposite (respectively, the same direction) to the waves, see [3]. This paradigm has been used innumerable times, for example in [4], which is one of the most popular attempts at reproducing the mechanisms of Taylor sheet in micrometre-sized artificial systems. In spite of this, there are still subtleties to be clarified when the amplitude of the deformations is large, including the inversion of the sign of the velocity, the possible trajectories and the characterization of optimal beats, e.g. [5,6].

Several variants of the concept of Taylor's swimming sheet have been proposed in the literature. Lauga and co-workers extended Taylor's series expansion of the solution to large amplitudes waves [7] and considered the case of sheet immersed in a viscolestic fluid [8]. Katz [9] studied the influence of the confinement on the resulting swimming speed, showing that in specific configurations the confinement can improve the swimming efficiency. The case of bending waves in cylinders was studied by Taylor himself [10] and more recently in [11], while [12] deals with an helicoidal geometry. Dasgupta *et al.* [13] report interesting experimental results on the case of a cylinder with a rotating wave of radius modulation in viscous and visco-elastic fluids.

In this paper, we propose a new rotational analogue of Taylor's sheet. Our analysis is motivated by the recent, and growing interest in the mechanics of shape-shifting structures [14–19]. We study a particular class of prestressed thin shells [18], and ask ourselves the question of what happens when these solid shells are immersed in a viscous fluid. As detailed later, these structures (which in the present case will be of circular or elliptical shape) attain, when actuated, a periodic pattern of shape-change, which is characterized by an almost constant curvature. Intuitively, the shell shape is almost cylindrical; its axis of maximal curvature performs a precession over time and the stored elastic energy is almost independent of its orientation. The shell is *neutrally stable* and its shape can be controlled by a weak embedded actuation [18]. The resulting deformation is the same as the one due to the propagation of a circular wave of displacement, transversal to the plane of the flat configuration of the shell. Thus, the shell can be seen as a rotational version of Taylor's swimming sheet. The goal of this paper is to address the question whether this device can be used as an artificial swimmer in a viscous fluid. In previous works on helical and cylindrical geometries, see [11,12], the use of symmetries and the fact that the structure is infinitely extended in one direction, allowed the authors for the effective reduction of the problem dimension. Instead, in the present case, the structure is finite, the shell deformation breaks the axial-symmetry, and we are forced to study a fully three-dimensional problem. We consider a simplified model for the shell. Assuming that the shell is inextensible and with uniform curvature, we retain a single degree of freedom, corresponding to the orientation of the direction of maximal curvature. In this framework, solving elementary Stokes problems with an adaptive finite element technique, we calculate the hydrodynamic coefficients modelling the interaction between the structure and the fluid. Hence, we solve the resulting fluid–structure interaction problem when either the structural shape or the actuating forces are prescribed.

In the following, §2 presents the mechanics of the neutrally stable shell, along with the relevant notation and the velocity fields generated under actuation. The swimming problem in a Stokes flow is formulated in §3, while §4 reports the numerical results. Conclusions are drawn in §5.

## 2. Neutrally stable cylindrical shells

Several recent works have shown that shell structures can exhibit a particularly rich behaviour thanks to the interplay between geometrical nonlinearities and pre-stresses. A basic example is an initially flat thin isotropic bimetallic disc $D:=\{X^{2}+Y^{2}<R^{2}\}$ of radius *R* subjected to a temperature loading. Because of the different expansion coefficients of the two layers composing the disc, a uniform temperature loading of the disc induces a uniform isotropic inelastic curvature, say $\bar{k}=\bar{c}\;I$, where **I** is the 2 × 2 identity tensor. As shown by [15,16,19,20], for sufficiently large $\bar{c}$, the disc can be modelled as an inextensible but flexible elastic surface, whose shape is characterized by an almost uniform curvature **k**. The inextensibility condition implies that the Gaussian curvature det(k) of the disc at the equilibrium remains equal to the initial Gaussian curvature, which is zero for an initially flat disc. Hence, the equilibrium shape should verify the condition det(k)=0. Under the uniform curvature assumption, this implies a cylindrical equilibrium shape with a curvature

**Figure 1.**
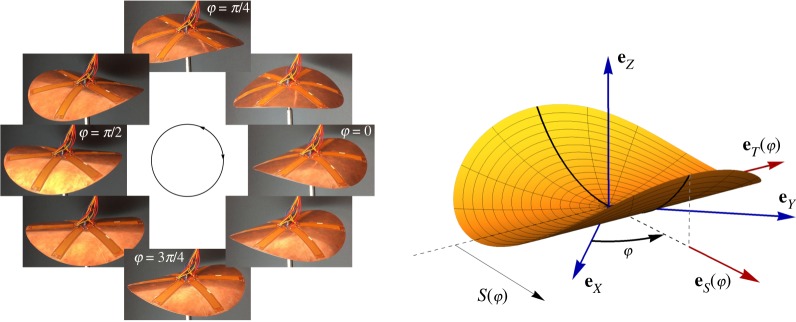
Shape-wave in the piezoelectric actuated neutrally stable shell. Left: experiments from [18]. Right: Kinematics and notation for the cylindrical shell used in this paper, where *φ* gives the rotation of the curvature axis.

2.1 $$k(c,\varphi )=c\;e_{S}(\varphi )⊗e_{S}(\varphi ),$$

where **e**_*S*_(*φ*) = cos*φ* **e**_*X*_ + sin*φ* **e**_*Y*_ is the axis of curvature, as sketched in figure 1, and the symbol ⊗ means the tensor product (***u***⊗***v***) · ***w*** = (***v*** · ***w***) ***u***. Here and henceforth, (*O*, **e**_*X*_, **e**_*Y*_, **e**_*Z*_) will denote an ortho-normal fixed reference frame, with **e**_*Z*_ orthogonal to the initial disc mid-plane D. For a perfectly isotropic disc, the elastic energy at the equilibrium is independent of the orientation *φ*, and reads as [18,19]

2.2 $$E(c,\varphi )=\frac{\pi R^{2}E\;h^{3}}{12(1-\nu^{2})}\left(\frac{c^{2}}{2}+(1+\nu )({\bar{c}}^{2}-c\;\bar{c})\right),$$

where *h* is the disc thickness and (*E*, *ν*) are its Young modulus and Poisson ratio. The stable equilibrium shapes of the disc are the minimizers of (2.2). For a given inelastic curvature $\bar{c}$, there is a continuous manifold of *neutrally stable* equilibria of curvature $c=(1+\nu )\bar{c}$, characterized by the same energy, independently of *φ*. Such a structure may be interesting for shape-control applications. Indeed, within the neutrally stable manifold, the shell can be deformed with vanishing actuation forces, while preserving a non-negligible stiffness for different deformation modes. This concept has been exploited in [18], by showing how to control a full precession of the axis of curvature of the disc with a weak multi-parameter piezoelectric actuation (figure 1). However, real structures are not perfectly isotropic. As shown in [18], slightly anisotropic discs are bistable and the actuation effort required to accomplish the full precession depends on a measure of their anisotropy.

The shape-change associated with the neutrally stable mode, i.e. the precession of the curvature axis, gives rise to a travelling wave where the velocity of each point of the disc is almost transversal to the mid-plane. This motion can be seen as the rotational analogue of the motion of Taylor's swimming sheet (1.1). To study the interaction of this structural motion with a surrounding fluid, we need a complete description of its kinematics, which is given below.

Let us denote by

2.3 $$X=Xe_{X}+Ye_{Y}\;\mathrm{such that}\;X^{2}+Y^{2}\leq R^{2},$$

the material points of the shell mid-plane in its flat reference configuration. For a curvature in the form (2.1), their current placement reads as

2.4 $$x=\chi \left(X;c,\varphi \right)=X+\left(\frac{\sin(c\;S(X,\varphi ))}{c}-S(X,\varphi )\right)e_{S}(\varphi )+\left(\frac{1-\cos(c\;S(X,\varphi ))}{c}\right)e_{Z},$$

where **e**_*S*_(*φ*) and **e**_*T*_(*φ*) are a pair of rotating orthonormal axes corresponding to the directions of maximal and vanishing curvatures, respectively, and

2.5 $$\begin{array}{rl} & S(X,\varphi )=X\cdot e_{S}(\varphi )=X\cos\varphi +Y\sin\varphi \\ \mathrm{and}\;\; & T(X,\varphi )=X\cdot e_{T}(\varphi )=-X\sin\varphi +Y\cos\varphi , \end{array}\}$$

are the coordinates of a material point **X** along these rotating axes, see figure 1 (right).

For a generic motion $x=\hat{x}(X,t)=\chi (X;c(t),\varphi (t))$, the Lagrangian description of the velocity field is given by

2.6 $$U_{s}(\text{X},t)=\frac{\partial \;\hat{x}(X,t)}{\partial t}={\frac{\partial \chi }{\partial c}|}_{t}\;\dot{c}(t)+{\frac{\partial \chi }{\partial \varphi }|}_{t}\;\dot{\varphi }(t),$$

where

2.7 $$\frac{\partial \chi }{\partial c}=\frac{c\;S\cos(c\;S)-\sin(c\;S)}{c^{2}}\;e_{S}+\frac{c\;S\sin(c\;S)+\cos(c\;S)-1}{c^{2}}\;e_{Z}=\frac{\;S^{2}}{2}e_{Z}-\frac{c\;\;S^{3}}{3}e_{S}+o(c)$$

and

**Figure 2.**
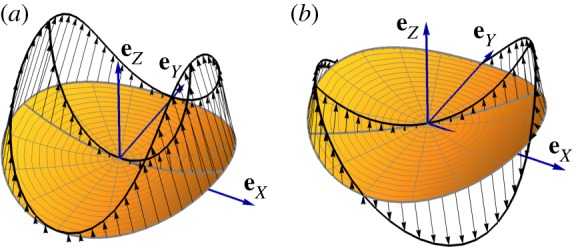
Velocity modes of the disc associated with variations of the curvature amplitude (*a*) and the curvature axis orientation (*b*), see equations (2.6)–(2.8).

2.8 $$\frac{\partial \chi }{\partial \varphi }=T\;(\cos(c\;S)-1)\;e_{S}+\left(\frac{\sin(c\;S)}{c}-S\right)\;e_{T}+T\sin(c\;S)\;e_{Z}=c\;S\;T\;e_{Z}+o(c),$$

are the velocity modes associated with variations of the curvature amplitude and the curvature axis orientation, respectively. Here *o*(*c*) means a quantity vanishing faster than *c* as *c* → 0. These velocity modes are sketched in figure 2.

In the following, we will study the fluid problem forced by these velocity fields on the moving surface of the disc. For the sake of simplicity, we will focus on the case where the curvature varies as in (2.1) with a constant *c*, which corresponds to the motion of a perfectly isotropic disc. The case of weakly anisotropic structures would introduce only minor perturbations to this motion, that we will ignore.

In the following, we will model the structure as a thick shell occupying in its reference configuration the three-dimensional domain $\Omega_{s}\equiv D\times [-h/2,h/2]$. We will denote by *Ω*_*s*_′ its deformed configuration under the action of the mapping (2.4). We will extend the mapping *χ* defined on the midplane D to *Ω*_*s*_ by using a nonlinear shell kinematics.

## 3. Stokes flow and forces from the fluid to the structure

### (a) Problem formulation

We study the interaction between the shell and a viscous fluid. It is assumed that the Reynolds number is low enough (*Re*≪1), and that the actuation is slow enough, such that the equations of motion for the fluid flow simplify to the (steady) Stokes equations. We neglect also the structural inertial effects and assume that the shell moves quasi-statically. As usual in fluid–structure problems, we adopt a Lagrangian description for the structure and a Eulerian description for the fluid. To correctly model the large displacement of the structure during the motion, we consider a geometrically nonlinear model.

We start by considering the case (i) where the shell deformation is assigned. In this framework, we compute the forces exerted by the fluid on the shell for several actuation conditions. This is a preliminary step to assess the potential behaviour of the neutrally stable shell with embedded actuation as a *pump* or a *swimmer*. Hence, we generalize these results to the case (§§4cii) of a coupled fluid–structure interaction. For a perfectly neutrally stable shell, we are able to solve a minimal version of the coupled problem giving a relationship between prescribed actuation forces and the resulting rotation speed.

#### (i) Swimming problem at imposed precession speed.

**Figure 3.**
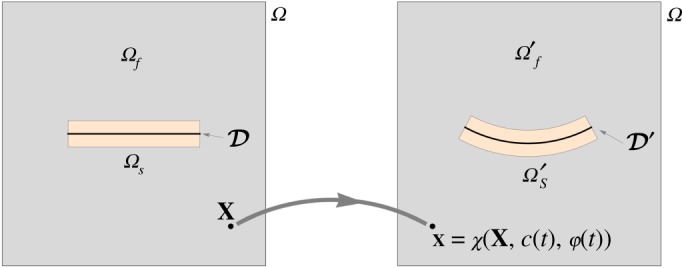
Structural (*Ω*_*s*_, *Ω*_*s*_′) and fluid (*Ω*_*f*_, *Ω*_*f*_′) domains in the reference and current configurations.

The computational domain for the fluid is taken as *Ω*_*f*_′ = *Ω*\*Ω*_*s*_′, where *Ω* is a closed box containing the fluid (figure 3). The case of an unbounded flow is approached for |*Ω*| → ∞. Hence, to determine the fluid flow for a given structural motion, we solve the Stokes equations for the fluid velocity **u** and the pressure *p* imposing the structural velocity field on the boundary between the fluid and the structure for a given shape *Ω*_*s*_′. They read as

3.1 $$\nabla p+△u=0,\;\nabla \cdot u=0\;\mathrm{in}\;\Omega_{f}^{\prime },$$

with the boundary conditions

3.2 $$u=u_{s}(x,t)\;\mathrm{on}\;\partial \Omega_{f}^{\prime }\cap \partial \Omega_{s}^{\prime }$$

and

3.3 $$u=0\;\mathrm{on}\;\partial \Omega_{f}^{\prime }\cap \partial \Omega .$$

The velocity field imposed on the boundary is the Eulerian version of the velocity field of the shell given in (2.6), superposed with a rigid motion:

3.4 $$u_{s}(x,t)=\dot{p}+\dot{\alpha }\times x+{\frac{\partial \chi }{\partial \varphi }|}_{X={\hat{x}}^{-1}(x,t)}\dot{\varphi },$$

where we assumed $\dot{c}=0$. Here, $\dot{p}$ is the velocity of the shell corresponding to a rigid translation of the centre of the disc and $\dot{\alpha }$ the axial vector associated with a rigid rotation. For *c* *R* < *π*, the inverse map ${\hat{x}}^{-1}(x,t)$ can be calculated explicity. For *φ* = 0, it reads as

$$S=\mathrm{sign}(x)\;\frac{\arccos(1-c\;z)}{c},\;T=y,\;Z=0,$$

and the Eulerian velocity field is

3.5 $${\frac{\partial \chi }{\partial \varphi }|}_{{\hat{x}}^{-1}(x,t)}=\mathrm{sign}(x)\left(\frac{\sqrt{(2-cz)cz}-\arccos(1-cz)}{c}e_{T}+y\sqrt{(2-cz)cz}\;e_{Z}\right)-c\;zy\;e_{S}.$$

The generic case *φ*≠0 can be obtained by a simple rotation around the *z*-axis.

Given the solution of the Stokes problem for an imposed velocity field on the boundary, one can evaluate the force and moment resultant of the stresses that the fluid exerts on the structure as follows:

3.6 $$F=\int_{\partial \Omega_{s}^{\prime }}\sigma n\;ds\;\mathrm{and}\;M=\int_{\partial \Omega_{s}^{\prime }}x\times \left(\sigma \;n\right)\;ds,$$

where ***σ*** = *p* **I** + 2*μ* (∇**u** + ∇**u**^T^) is the Cauchy stress in the fluid, ***n*** is the unit normal pointing inside the fluid domain, *ds* denotes the surface measure and ***x*** is the position vector of the generic point in the current configuration. We take the centre of disc ***o*** as the origin and as the pole for the moment ***M***. Because of the linearity of the Stokes equations and the linearity of the velocity field (3.4) imposed on the boundary with respect to ($\dot{p},\dot{\alpha },\dot{\varphi }$), also (***F***, ***M***) will depend linearly on ($\dot{p},\dot{\alpha },\dot{\varphi }$). Hence, we can write

3.7 $$\left(\begin{array}{c} F\\ M \end{array}\right)=K\left(\begin{array}{c} \dot{p}\\ \dot{\alpha } \end{array}\right)+\left(\begin{array}{c} k_{p\varphi }\\ k_{\alpha \varphi } \end{array}\right)\dot{\varphi }\;\mathrm{and}\;K:=\left(\begin{array}{cc} K_{pp} & K_{p\alpha }\\ K_{p\alpha }^{T} & K_{\alpha \alpha } \end{array}\right),$$

where K is a symmetric negative-definite 6 × 6 matrix, because of the dissipative nature of the Stokes equations. The components of the columns of K can be computed by solving six problems where only one of the components of $\dot{p}$ or $\dot{\alpha }$ is set to one, while the other are set to zero, and evaluating the corresponding force and moment resultants with (3.6). Similarly, the two 3 × 1 vectors **k**_***p****φ*_ and **k**_***α****φ*_ are computed by solving the Stokes problem with $\dot{p}=\dot{\alpha }=0$ and $\dot{\varphi }=1$.

If the body is completely unconstrained, one can deduce the instantaneous free *swimming* velocity of the shell by imposing the quasi-static equilibrium conditions ***F*** = **0**, ***M*** = **0**, giving

3.8 $$\left(\begin{array}{c} \dot{p}\\ \dot{\alpha } \end{array}\right)=-K^{-1}\left(\begin{array}{c} k_{p\varphi }\\ k_{\alpha \varphi } \end{array}\right)\dot{\varphi }.$$

Assuming that the resultants on the body vanish corresponds to the *zero-thrust* velocity-generating function of the swimmer according to Lighthill [21]. Here, we study the relevant particular case where the shell rigid motion is the composition of a translation along the *Z*-axis, controlled by $\dot{p}$, and a rotation around the same axis, controlled by $\dot{\alpha }$

3.9 $$\dot{p}=\dot{p}\;e_{Z}\;\mathrm{and}\;\dot{\alpha }=\dot{\alpha }\;e_{Z}.$$

The constraints are supposed to be perfect so that

3.10 $$F\cdot e_{Z}=0\;\mathrm{and}\;M\cdot e_{Z}=0.$$

Using (3.7), (3.9) and (3.10), we obtain the equations for the constrained swimming as

3.11 $$\left(\begin{array}{cc} k_{pp} & k_{p\alpha }\\ k_{p\alpha } & k_{\alpha \alpha } \end{array}\right)\left(\begin{array}{c} \dot{p}\\ \dot{\alpha } \end{array}\right)+\left(\begin{array}{c} k_{p\varphi }\\ k_{\alpha \varphi } \end{array}\right)\dot{\varphi }=\left(\begin{array}{c} 0\\ 0 \end{array}\right),$$

where

3.12 $$\begin{array}{rl} & k_{pp}=K_{pp}e_{Z}\cdot e_{Z},\;k_{p\alpha }=K_{p\alpha }e_{Z}\cdot e_{Z},\;k_{\alpha \alpha }=K_{\alpha \alpha }e_{Z}\cdot e_{Z}\\ \mathrm{and}\;\; & k_{p\varphi }=k_{p\varphi }\cdot e_{Z},\;k_{\alpha \varphi }=k_{\alpha \varphi }\cdot e_{Z}. \end{array}\}$$

The coupling coefficient of the resistance matrix *k*_*pα*_ relates the coupling between the rigid body translation and rotation of the structure, and is associated with the chirality of the structural shape [2]. From a dimensional analysis of the Stokes problem, we can deduce that the relevant coefficients can be written as follows:

3.13 $$\begin{array}{rl} & k_{pp}=\mu R\;{\hat{k}}_{pp}(c,\varphi ),\;k_{p\alpha }=\mu R^{2}\;{\hat{k}}_{p\alpha }(c,\varphi ),\;k_{\alpha \alpha }=\mu R^{3}\;{\hat{k}}_{\alpha \alpha }(c,\varphi )\\ \mathrm{and}\;\; & k_{p\varphi }=\mu R^{2}\;{\hat{k}}_{p\varphi }(c,\varphi ),\;k_{\alpha \varphi }=\mu R^{3}\;{\hat{k}}_{\alpha \varphi }(c,\varphi ), \end{array}\}$$

where ${\hat{k}}_{ij}$ are dimensionless scalar functions of the two shape parameters (*c*, *φ*) of the disc.

#### (ii) Swimming problem at imposed actuation forces.

In §2, we presented an inextensible uniform-curvature structural model where the shell is described by two degrees of freedom: the curvature amplitude *c* and direction *φ*. For an almost neutrally stable shell, the stiffness with respect to *c* is much higher than the one with respect to *φ*. As far as the inextensible shell model is pertinent, this stiffness ratio is vanishing independently of the shell thickness and Young modulus, see [17,18]. Hence, we will study the coupled fluid–structure interaction under the further approximation that *c* is constant, and *φ* is the only structural degree of freedom left to describe the shell deformation.

In this framework, the shell equilibrium equation is written as

3.14 $$k_{p\varphi }\dot{p}+k_{\alpha \varphi }\dot{\alpha }+k_{\varphi \varphi }\dot{\varphi }+\frac{\partial E(c,\varphi )}{\partial \varphi }=\bar{M},$$

where $\bar{M}$ is a ‘twisting’ moment modelling the effect of the embedded actuation, see [18], and *k*_*φφ*_ is the drag on the precession motion. This last coefficient can be computed by evaluating the tractions ***σn*** for the Stokes problem with $\dot{p}=\dot{\alpha }=0$ and $\dot{\varphi }=1$ and projecting them on the velocity field (2.8), i.e.

3.15 $$k_{\varphi \varphi }=\int_{\partial \Omega_{s}^{\prime }}\sigma n\cdot {\frac{\partial \chi }{\partial \varphi }|}_{{\hat{x}}^{-1}(x,t)}\;ds.$$

The system obtained coupling (3.14) with (3.11), is a minimal version of the coupled fluid–structure problem at imposed actuation forces. Its solution allows us to determine the swimming speeds $\dot{p}$ and $\dot{\alpha }$ as a function of the applied moment $\bar{M}$. Remark that for a perfect neutrally stable shell the elastic force ∂E(c,φ)/∂φ vanishes, as the elastic energy E(c,φ) in (2.2) does not depend on the curvature direction *φ*. This fact will be exploited in §4cii.

Equation (3.14) can be extended to account for further structural deformation modes (e.g. *c*), and inertial effects. However, this is out of the scope of the present work. The rest of the paper is devoted to determine the coefficients in (3.13) and understand the key properties of the flow generated by the shell deformation. This will allows us to deduce the properties of the neutrally stable shells as swimmers, when driving the precession of the curvature axis with an embedded actuation.

### (b) Numerical methods

To obtain the forces and torques acting on the immersed shell, we follow the procedure reported below:

(i) Generate the mesh for the holed fluid domain, *Ω*_*f*_′ = *Ω*\*Ω*_*s*_′, where *Ω*_*s*_′ is the deformed configuration of the shell, see figure 3-left.
(ii) Solve the outer Stokes problem on the deformed domain *Ω*_*f*_′ forced by the Eulerian velocity field (3.5) on the solid boundary. We use an adaptive finite-element solver, based on a *a posteriori* error indicator.
(iii) Integrate, using (3.6), the stresses on the boundary ∂*Ω*_*s*_′∩∂*Ω*_*f*_′ to obtain the resultant force and moment exerted by the fluid on the shell.

The holed domain *Ω*_*f*_′ is discretized with tetrahedral elements using the mesh generator gmsh [22]. The Stokes problem is discretized and solved with standard finite-elements techniques, implemented through the FEniCS [23] framework in python language. Using a solid domain with finite thickness allows us to represent different values of the stresses on the top and bottom shell surfaces without resorting to more involved numerical methods, such as Discontinuous Galërkin for the finite-element approximation. We adopt a Taylor–Hood discretization of the displacement and pressure field on piecewise quadratic and piecewise linear Lagrange finite elements, respectively. Hence, we use iterative solvers and pre-conditioners provided by PETSc [24] to solve the underlying saddle problem on multiple processors. Iterative mesh adaptation is performed using a standard *a posteriori* error estimator for the Stokes problem [25].

**Figure 4.**
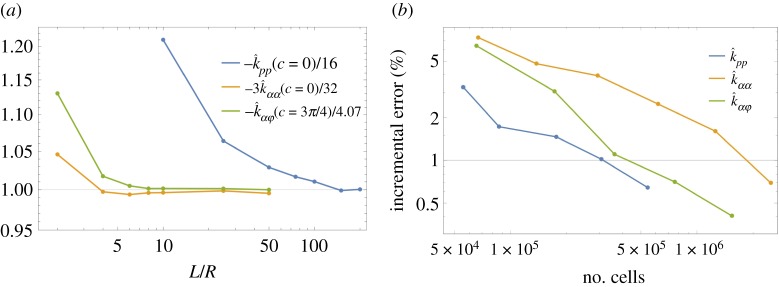
Numerical convergence of the hydrodynamic coefficients in (3.13) for a circular shell. (*a*) Convergence with respect to the size *L* of the bounding box *Ω*; ${\hat{k}}_{pp}(c=0)$ and *k*_*αα*_(*c* = 0) have been scaled with respect to their analytical values, while ${\hat{k}}_{\alpha \varphi }$ is scaled with respect to its numerical value when *L* = 200*R*. (*b*) Convergence with respect to the number of FE cells for *L* = 100 *R*.

The simulation is performed on a cylindrical box *Ω* = {|*Z*| < *H*,  (*X*^2^ + *Y*^2^) < *L*^2^}. The size of the mesh and the size of the bounding box *Ω* are set to correctly reproduce the results of analytical solutions, in special cases in which such solutions are available. The influence of the size of the box is shown in figure 4*a*, which reports the drag coefficients *k*_*pp*_(*c*) and *k*_*αα*_(*c*) for the case of a flat disc *c* = 0. The numerical results converge to the analytic estimates for unbounded domains, respectively ${\hat{k}}_{pp}(c)=-16$ and ${\hat{k}}_{\alpha \alpha }(c)=-32/3$, see [26,27]. The results of figure 4 are obtained on a cylindrical box of eight *H* = 100 *R*.

Figure 4*b* shows the percentage incremental error with respect to the total number of finite-element cells. In the numerical simulations, we use an adaptive mesh refinement based on the *a posteriori* error estimate suggested in [25]. Once the total error is summed over all the cells, only the ones having percentage error higher than a fixed fraction are refined using the method of [28]. This process is iterated until the evaluation of the forces and moments on the shell converge within a 0.5% variation with respect to the previous step.

Overall, considering the errors due the discretization and the finite size of the box, we can safely assume that the results produced in the rest of this paper are accurate within an error margin of 1%.

## 4. Results

This section presents the results of the numerical simulations, performed to calculate the hydrodynamic coefficients in equation (3.11). Both the cases of swimming at imposed speed and at imposed actuation are considered. Hence, we rationalize the key features of the flow generated by the shell deformation. We will consider first the case of a circular shell. In the last subsection, we will present the results for the elliptic case.

### (a) Hydrodynamics coefficients for a circular shell

We solve numerically three Stokes problems defined by the following velocity fields on the fluid–structure interface:

(i) Rigid body translation in the *Z*-axis direction: $\dot{p}=1,$ $\dot{\alpha }=\dot{\varphi }=0$ to compute *k*_*pp*_ and *k*_*pα*_;
(ii) Rigid body rotation around the *Z*-axis: $\dot{\alpha }=1,$ $\dot{p}=\dot{\varphi }=0$ to compute *k*_*αα*_;
(iii) Precession of the curvature axis: $\dot{\varphi }=1,$ $\dot{p}=\dot{\alpha }=0$ to compute *k*_*pφ*_, *k*_*αφ*_ and *k*_*φφ*_.

The hydrodynamics coefficients (3.13) are computed by evaluating the associated force and moment resultants as in (3.6). These coefficients generally depend on the amplitude, *c*, and orientation, *φ*, of the shell curvature. In the case of a shell with a circular flat reference configuration, the symmetries of both the geometry and the loading imply the following simplifications:

— All the hydrodynamics coefficients are independent of *φ*. Without loss of generality, one can set *φ* = 0. This is tantamount to choose the reference frame {*O*, **e**_*S*_, **e**_*T*_, **e**_*Z*_} in (3.11).
— The coupling coefficient *k*_*pα*_(*c*) vanishes for any value of *c*. This coefficient, being the torque resultant for an imposed rigid translation $\dot{p}\;e_{Z}$, vanishes because the shell shape and the load are invariant under reflections with respect to **e**_*T*_–**e**_*Z*_ and **e**_*S*_–**e**_*Z*_ planes.
— The pumping force resultant *k*_*pφ*_(*c*) vanishes for any curvature *c*. This is due to the symmetries of the shell shape and the following properties of the velocity field (2.6): *U*_*S*_(*S*, *T*) = *U*_*S*_( − *S*, *T*) = − *U*_*S*_(*S*, − *T*), *U*_*T*_(*S*, *T*) = − *U*_*T*_( − *S*, *T*) = *U*_*T*_(*S*, − *T*), *U*_*Z*_(*S*, *T*) =  − *U*_*Z*_( − *S*, *T*) =  − *U*_*Z*_(*S*, − *T*), where *U*_*i*_ = ∂_*φ*_***χ*** · ***e***_*i*_.

**Figure 5.**
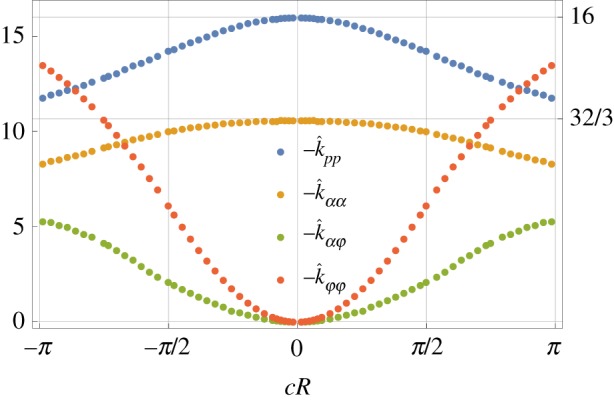
Coefficients of hydrodynamic resistance (3.13) as functions of the curvature *c* of a circular shell; ${\hat{k}}_{\varphi \varphi }=\mu R^{3}k_{\varphi \varphi }$ is the dimensionless form of the drag (3.15).

Figure 5 reports the dimensionless version of the hydrodynamics coefficients ${\hat{k}}_{pp}(c)$, ${\hat{k}}_{\alpha \alpha }(c)$, ${\hat{k}}_{\alpha \varphi }(c)$ and ${\hat{k}}_{\varphi \varphi }(c)$ as a function of the dimensionless curvature *c* *R*. These plots are universal and independent of any physical parameter. The values reported here are computed on domains sufficiently large to neglect the effect of the bounding box *Ω*, *L* = 200*R* in figure 4.

**Figure 6.**
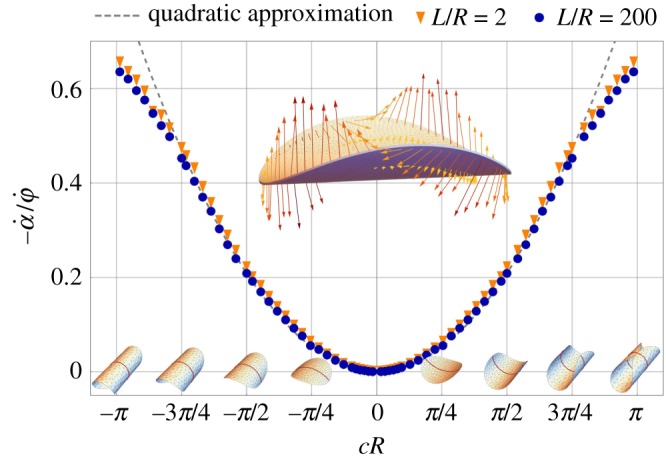
Rotational swimming velocity $\dot{\alpha }$ for a circular shell generated by the precession velocity of the curvature axis $\dot{\varphi }$; blue dots, numerical results for a cylindrical box of radius *L*/*R* = 200; orange triangles, numerical results for a box of radius *L*/*R* = 2; dashed line, quadratic approximation for moderate curvature (4.3). Inset: fluid-to-structure net contact forces distribution for *cR* = 0.8 as found by extracting from the numerical simulation the jump of the contact force between the upper (+) and lower (–) shell surfaces, ***σn*** = ***σ***^+^***n***^+^ − ***σ***^−^***n***^−^.

The value of *k*_*pφ*_(*c*), not reported in figure 5, turns out to be zero at the numerical accuracy, as anticipated above. A simple intuitive justification is the following: the precession of the axis of curvature causes (mainly) different sectors of the disc to move upwards or downwards in the direction that is perpendicular to the plane of the flat disc. Due to the geometry of the shell, the area of the sectors of the disc that move up or down is identical and therefore no net momentum flux is generated, and thus the integral of stresses over the shell should be zero. This is visually confirmed by inspecting the fluid-to-structure contact forces distribution reported in the top inset of figure 6. They respect the same symmetries of the components *U*_*i*_ of the velocity field imposed on the boundary.

### (b) Swimming problem at imposed precession speed $\dot{\varphi }$

Applying in (3.11) the simplifications due to the symmetry reported above, one can readily solve the swimming problem at imposed precession speed $\dot{\varphi }$ to get

4.1 $$\dot{p}(c)=-\frac{k_{p\varphi }(c)}{k_{pp}(c)}\;\dot{\varphi }=0\;\mathrm{and}\;\dot{\alpha }(c)=-\frac{k_{\alpha \varphi }(c)}{k_{\alpha \alpha }(c)}\;\dot{\varphi }.$$

The *swimming* motion of the shell is a pure rotation around the *Z*-axis with a speed $\dot{\alpha }$ driven by a non-vanishing torque resultant *k*_*αφ*_ in (4.1).

Using the numerical values reported in figure 5, figure 6 summarizes our findings by plotting the ratio between the swimming rotation speed $\dot{\alpha }(c)$ and the driving speed $\dot{\varphi }$ of the deformation wave as function of the dimensionless shell curvature *cR*. The rotational swimming motion driven by the precession of the shell curvature axis is the rotational analogue of the translation swimming motion of Taylor's sheet [1]. The deformation of the shell can be regarded as a circular travelling wave of transversal displacement.

For a Taylor's sheet, the ratio between the translational swimming velocity *U* and the phase speed *v* of the deformation wave is (1.4)

4.2 $$\frac{U}{v}=-\frac{1}{2}{\left(b\;k\right)}^{2},\;\mathrm{for}\;|b\;k|\ll 1.$$

In our case, the wavenumber is fixed to *k* = 2/*R*, while, in the shallow shell approximation, the wave amplitude scales as *b*∝*c* *R*^2^. Substituting these relations in (4.2), as *b* *k*∼*c* *R*, the rotational velocity is expected in the form $\dot{\alpha }/\dot{\varphi }\propto -{\left(c\;R\right)}^{2}$.

This is confirmed numerically in figure 6, which shows the swimming angular speed produced for a symmetric range of curvatures. The numerical data may be fitted by the following quadratic approximation in *c* *R*:

4.3 $$\frac{\dot{\alpha }(c)}{\dot{\varphi }}≃-\frac{1}{12.0}{\left(c\;R\right)}^{2}\;\mathrm{for}\;|c\;R|≲\frac{3\pi }{4}.$$

This approximation is the analogue of the Taylor formula (4.2) for a flat sheet. Our numerical results show that the quadratic approximation is accurate even for *c* *R*≃1. Here, the dimensionless parameter *c* *R* measures the central angle of the cylindrical configuration of the shell. As shown in the lower insets of figure 6 for *c* *R* = *π*, its cross section with the plane *T* = 0 is a circle. Interestingly, the efficiency of the shell as a rotational swimmer is very robust both with respect to the curvature amplitude and the radius of the confining box. The numerical results for a cylindrical bounding box of radius *L*/*R* = 2 and *L*/*R* = 200 are almost indistinguishable and close to the quadratic approximation even for |*c* *R*| → *π*. The height of the cylinder is fixed here to *H*/*R* = 100.

### (c) Swimming problem at imposed actuation

The swimming problem at imposed actuation consists in solving the system (3.11) and (3.14) for $\dot{p}$, $\dot{\alpha }$ and $\dot{\varphi }$ as functions of $\bar{M}$.

In general, this is a system of ordinary differential equations in time, but, for a perfectly neutrally stable shell, the elastic force ∂E(c,φ)/∂φ in (3.14) vanishes. Hence, the system reduces to a linear algebraic system in the velocities $(\dot{p},$ $\dot{\alpha },$ $\dot{\varphi })$. Moreover, for the case of circular shells, the symmetries imply *k*_*pα*_ = *k*_*pφ*_ = 0 for any value of the curvature *c*, see §4a; the system simplifies to

4.4 $$\left(\begin{array}{ccc} k_{pp}(c) & 0 & 0\\ 0 & k_{\alpha \alpha }(c) & k_{\alpha \varphi }(c)\\ 0 & k_{\alpha \varphi }(c) & k_{\varphi \varphi }(c) \end{array}\right)\left(\begin{array}{c} \dot{p}\\ \dot{\alpha }\\ \dot{\varphi } \end{array}\right)=\left(\begin{array}{c} 0\\ 0\\ \bar{M} \end{array}\right),$$

which can be readily solved to get

4.5 $$\dot{p}=0,\;\dot{\alpha }=\frac{k_{\alpha \varphi }(c)\;\bar{M}}{k_{\alpha \varphi }^{2}(c)-k_{\varphi \varphi }k_{\alpha \alpha }(c)}\;\mathrm{and}\;\dot{\varphi }=\frac{-k_{\alpha \alpha }(c)\bar{M}}{k_{\alpha \varphi }^{2}(c)-k_{\varphi \varphi }(c)k_{\alpha \alpha }(c)}.$$

**Figure 7.**
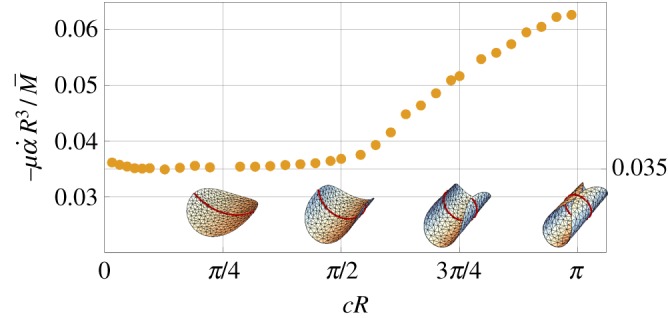
Swimming rotation speed $\dot{\alpha }$ for applied moment $\bar{M}$ as a function of the dimensionless curvature *c* *R* of a circular shell. The dots are computed from (4.5)_2_ for the data in figure 5. The value 0.035 has been estimated by a quadratic approximation of the hydrodynamic coefficients near *c* = 0.

The most interesting result is the ‘swimming’ rotation speed $\dot{\alpha }$ generated by the actuation moment $\bar{M}$, which is plotted in figure 7 using the numerical values of the hydrodynamic coefficients reported in figure 5. We observe two regimes: for *c* *R*≤*π*/2 the ratio $\dot{\alpha }/\bar{M}$ is almost independent of the curvature while for *c* *R* > *π*/2 the swimming efficiency grows almost linearly with the curvature. We do not have a clear explanation for this result; however, we can observe that after the curvature value *c* *R* = *π*/2 the shell starts curling up and tends to a closed cylindrical shape, see the insets in figure 7. It is therefore reasonable to expect a qualitative difference in the dependence of the hydrodynamic coefficients on the curvature in the two regimes. Indeed, *c* *R* = *π*/2 corresponds to an inflection point for the hydrodynamic coefficients in figure 5.

### (d) Structure of the fluid flow (circular case)

**Figure 8.**
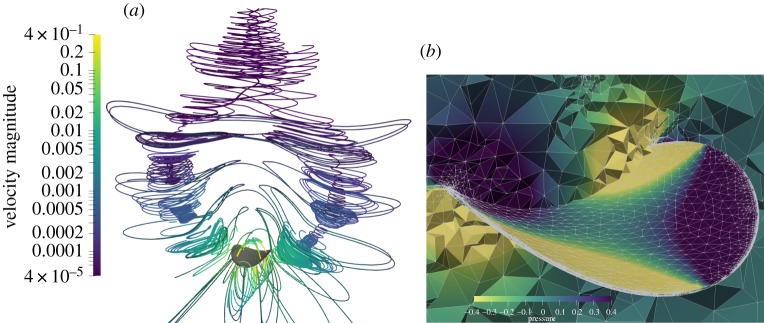
Structure of the flow generated by the precession of the shell curvature axis with *c* = 0.8 and *R* = 1 for the loading case (iii). (*a*) Streamlines with the shell in grey. (*b*) Illustration of the mesh after adaptive refinement used for the 3D finite-element calculations; the colours represent the pressure field.

We now analyse the structure of the fluid flow driven by the precession, $\dot{\varphi }=1$, of the curvature axis when the shell is clamped ($\dot{\alpha }=0$, $\dot{p}=0$) and, hence, acts as a *pump*. Figure 8 reports the stream lines (*a*) and the pressure distribution (*b*) in a small region around the shell for the loading case (iii) and *c* = 0.8/*R*. The near-field flow (close to the structure) shows four vortices emanating from the shell in the directions of maximal (*S*) and vanishing (*T*) curvatures. The two vortices in the direction of maximal curvature bend upwards following the curvature of the shell. At distance *Z*∼10*R*, they coalesce into a single vortex on the top of the shell, giving the far-field structure of the flow for *Z* → + ∞.

**Figure 9.**
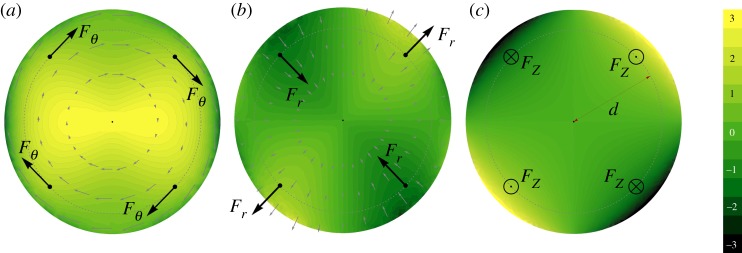
Decomposition of the net contact force field (see the inset of figure 6) in the circumferential component *F*_*θ*_ (*a*), the radial component *F*_*r*_ (*b*) and the pressure *p* (*c*). The black arrows are a system of four forces reproducing the same symmetry, and used in the far-field approximation (4.8).

The key properties of the flow can be rationalized in terms of fundamental solutions of the Stokes equations to point forces. To this end, we project the fluid-to-structure net contact forces, represented in the inset of figure 6, on the Gauss frame associated with the shell mid-surface ***a***_*r*_, ***a***_*θ*_, ***n***. Here, ***a***_*r*_ and ***a***_*θ*_ are the radial and circumferential tangent unit vectors, respectively, and ***n*** = ***a***_*r*_ × ***a***_*θ*_ is the normal. The components of the net contact force field *F*_*r*_ = ***σn*** · ***a***_*r*_, *F*_*θ*_ = ***σn*** · ***a***_*θ*_, and *F*_*n*_ = ***σn*** · ***n*** are plotted in figure 9. The far-field effect of this force field on the fluid flow can be reproduced by four equivalent point forces, one for each quadrant of the shell. To this aim, within the shallow shell approximation, we fix a cylindrical coordinate system (*O*, ***e***_*r*_(*θ*), ***e***_*θ*_(*θ*), ***e***_*Z*_) in the *X*–*Y* plane. The equivalent four forces, respecting the same symmetries of the net contact force distribution, are (*i* = 1, …, 4)

4.6 $$F_{i}=F_{\theta }e_{\theta }(\theta_{i})+\mathrm{sign}(\tan\theta_{i})(F_{r}e_{r}(\theta_{i})+F_{Z}e_{Z}),\;i=1,\ldots 4$$

applied at points ***x***_*i*_ = *d* ***e***_*r*_(*θ*_*i*_) with *θ*_*i*_ = (2*i* − 1)*π*/4 (points on the bisector lines at distance *d*∼*R* from the centre, see the black arrows in figure 9). The velocity field generated by a point unit force applied at the point ***y*** in a direction ***e*** is

4.7 $$G(x,y,e)=\frac{e}{∥x-y∥}+\frac{e\cdot (x-y)}{∥x-y{∥}^{3}}(x-y)$$

usually called *Stokeslet*. Hence, the far-field approximation velocity field generated by the pattern of the four point forces is calculated by superposing the corresponding *Stokeslet* and taking the series expansion for *d*≪1. We get

4.8 $$v(x)=\sum_{i=1}^{4}G(x,x_{i},F_{i})=d\;(F_{\theta }R(x)+F_{r}\;S(x))+F_{Z}\;Q(x)\;\frac{d^{2}}{2}+o(d^{3}),$$

with

**Figure 10.**
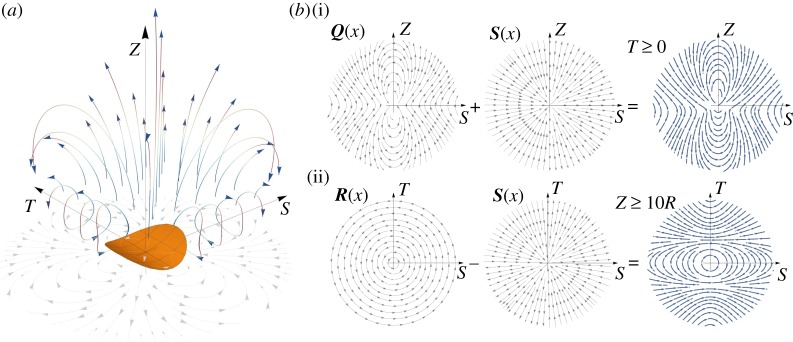
Qualitative representation of the velocity field by singular solutions, see (4.8) and (4.9). (*a*) Streamlines of the quadrupole term ***Q***. (*b*) Effects of the superposition of a quadrupole ***Q*** and a stresslet ***S*** in the plane 0 < *T*≪1 (i) and of a rotlet ***R*** and a stresslet in the plane *Z*≫1 (ii).

4.9 $$R(x)=\frac{e_{Z}\times \hat{x}}{∥x{∥}^{2}},\;S(x)=\frac{\sin(2\theta )}{2}\frac{\hat{x}}{∥x{∥}^{2}}\;\mathrm{and}\;Q(x)=\frac{q(\hat{x})}{∥x{∥}^{3}},$$

where $\hat{x}=x/∥x∥$. As the four Stokeslets are distributed on a surface, due to the symmetry of the problem, the lowest-order contribution in the resulting singular velocity field (4.8) is a dipole decaying as 1/∥***x***∥^2^. The Stokeslet term, decaying as 1/∥***x***∥ is instead vanishing. The dipole can be decomposed in a skew-symmetric part, the *rotlet* ***R***, and a symmetric part, the *stresslet* ***S***. The higher order term in the expansion (4.9) is a *quadrupole* ***Q***, see [26], decaying as 1/∥***x***∥^3^. The explicit analytical expression of $q(\hat{x})$ is available in classical textbooks [29]; we report in figure 10 the corresponding streamlines instead. The field close to the shell surface is dominated by the quadrupole term with its four vortices. Vice-versa the far-field is dominated by the rotlet and stresslet terms. The streamlines resulting by the superposition of ***R***, ***S*** and ***Q*** are sketched in figure 10, which qualitatively reproduces the main structures observed in the direct numerical simulation of figure 8.

### (e) The case of elliptic shells

**Figure 11.**
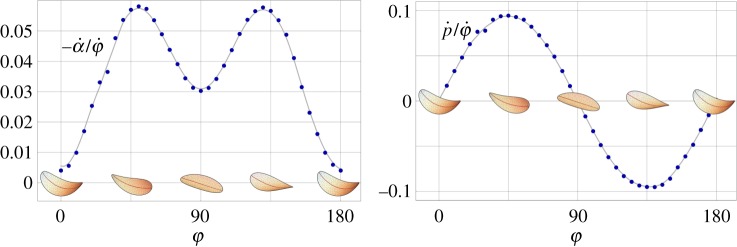
Rotational ($\dot{\alpha }$) and translational ($\dot{p}$) velocities of an elliptic shell during the precession of its curvature axis (*φ*): results of a numerical simulation on a shell with semi-axes *R*_*X*_ = 1 and *R*_*Y*_ = 1/2, and curvature *c* = 0.8/*R*_*X*_ computed using (3.11).

In the circular case, the force resultant *k*_*pφ*_ and the coupling coefficient *k*_*pα*_ vanish because of the symmetries. For a shell with an elliptical shape in its flat reference configuration, these coefficients are non-null. Moreover, all the coefficients in (3.11)–(3.13) depend on the direction of maximal curvature *φ*. We report here the results obtained from the numerical simulation for a shell with semi-axes *R*_*X*_ = 1 (*X* direction) and *R*_*Y*_ = 1/2 (*Y* direction) and a curvature amplitude *c* = 0.8/*R*_*X*_. Figure 11 plots the translational and rotational swimming velocities, $\dot{p}$ and $\dot{\alpha }$ as a function of the orientation of the curvature axis *φ*∈(0, 2*π*), during its full precession. In this case, the translation swimming velocity is non-null and the rotational velocity varies during a period. However, the translation velocity (and displacement) is periodic, the shell moves back and forth on the *Z*-axis while rotating, but there is not a net translation after an integer number of periods. This can be seen as a direct consequence of the *scallop theorem* [30]. The video attached in the electronic supplementary material helps to visualize this case.

## 5. Conclusion

**Figure 12.**
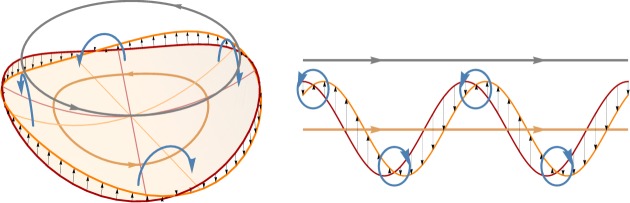
Schematics of the basic mechanism of the rotational Taylor sheet, extending to the rotational shape wave of neutrally stable disc (left) the physical interpretation of [2] of the classical Taylor sheet with a translation shape wave. We report in red and orange the configuration of the structure at time *T* and *T* + Δ*T*, with small Δ*T*, in a reference frame moving with the structure, where the black arrows indicate the velocity field of the structure and the blue arrows schematically represent the vorticity in the fluid. The grey arrows indicate the flow velocity at infinity, which is opposite to the swimming velocity in a fixed reference frame.

It has been shown how the problem of a complex active structure exhibiting spontaneous curvature, moving within a (viscous) fluid, can be connected to classic examples of locomotion at low *Reynolds* numbers, in particular, the work of Taylor [1]. The main issue in this setting is that linearity of Stokes equations results in the celebrated observation due to Purcell [30], usually referred to as the *scallop theorem:* a reciprocal motion (precisely, a shape-change sequence that is identical after time-reversal [2]) does not generate net motion on average. The scallop theorem implies that the swimming strategy has to satisfy some geometrical requirements in order to be effective. The precession of the curvature axis can be seen as a travelling (circular) wave of transversal displacement, similar to the case of the Taylor sheet. This analogy is summarized in figure 12, which transposes to our case the kinematical interpretation of Taylor's sheet effect given in [2]. The shape wave generates a system of counter-rotating vortices resulting in a net rotational motion of the swimmer. In the circular case, the symmetry of the problem prevents net translations. Interestingly, if instead the shape of the shell in the flat configuration is elliptic, a net force, and thus instantaneous displacement is generated. The force however is periodic and therefore the displacement is zero over a period.

Directions of further research might involve relaxing the hypothesis of very low *Reynolds* numbers, in order to consider cases when inertia is no longer negligible. This might include exploiting instabilities of thin structures, as bistable clamped tails [14], or pulsatile motions [31]. In this framework, the elastic response of the structure would play a crucial role and can be accounted for by using reduced models or finite-element shell models [32], coupled with a Navier–Stokes solver.

Other interesting applications include the possibility of using the neutrally stable shells studied in this paper to harvest energy from the fluid flow [33,34] or as deformable mixing devices.

## Supplementary Material

fzerostiffnesscircle.m4v

## Supplementary Material

fzerostiffnessellipse.m4v

## Data accessibility

All the data in the paper can be generated by running the FEniCS codes found in the repository https://github.com/cmaurini/stokes-shells. These codes are freely accessible. FEnics is an open-source FE framework that can be obtained here https://fenicsproject.org/download.
